# Pseudocirrhosis caused by lung adenocarcinoma with diffuse liver metastasis: An autopsy case report

**DOI:** 10.1111/1759-7714.14010

**Published:** 2021-05-19

**Authors:** Sachiko Nakano, Tsukasa Suzuki, Yoshiaki Takase, Masashi Ito, Takashi Osaki, Akihiro Yoshii, Takashi Terauchi

**Affiliations:** ^1^ Department of Nuclear Medicine Cancer Institute Hospital Koto Tokyo Japan; ^2^ Department of Diagnostic Radiology Shibukawa Medical Center Shibukawa Gunma Japan; ^3^ Department of Pathology Shibukawa Medical Center Shibukawa Gunma Japan; ^4^ Department of Thoracic Surgery Sapporo Medical University Hospital Sapporo Hokkaido Japan; ^5^ Department of Respiratory Medicine Shibukawa Medical Center Shibukawa Gunma Japan

**Keywords:** liver, lung adenocarcinoma, lymph node metastasis

## Abstract

We describe a rare case of a 64‐year‐old man with lung adenocarcinoma with lymph node and bone metastases who developed pseudocirrhosis. Initial examination revealed a hepatic disorder of unknown cause with narrowing of the portal vein and a low‐density area surrounding the portal veins in computed tomography (CT) imaging. Diffuse liver metastasis was diagnosed after percutaneous liver biopsy. During chemotherapy, liver atrophy and irregular liver surface appearance were confirmed with CT. Eventually, the disease progressed to death, and an autopsy was performed. The autopsy demonstrated exacerbation of diffuse liver metastases and cirrhosis‐like findings.

## Graphical abstract

Even in lung adenocarcinoma, if liver damage with abnormal imaging findings around the portal vein is observed, diffuse liver metastasis can be suspected. A take‐away message from this report is that pseudocirrhosis can occur in lung cancer as well.

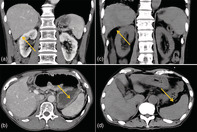

## INTRODUCTION

Pseudocirrhosis has imaging findings similar to those of cirrhosis without the characteristic pathological features of cirrhosis.^1^ It occurs frequently in breast cancer and is said to occur with liver metastasis.^2^ Pseudocirrhosis can be said to be a pathological condition, in which other causes of cirrhosis are excluded and liver metastasis is present in the background and cirrhosis is also present. There are a few reports on the occurrence of pseudocirrhosis with thyroid cancer, pancreatic cancer, gastric cancer, and colon cancer.^3^, ^4^, ^5^, ^6^ However, to our knowledge, there is only one report on pseudocirrhosis caused by oat cell lung cancer in English literature.^7^ Further, diffuse liver metastasis has been reported in small cell lung cancer^7^; however, to the best of our knowledge, there are no reports on diffuse liver metastasis of lung adenocarcinoma.

We report an autopsy case of pseudocirrhosis associated with diffuse liver metastasis of lung adenocarcinoma.

## CASE PRESENTATION

A 64‐year‐old male was admitted to our hospital with a complaint of a cough and shortness of breath. On admission, the patient's heart rate (HR) was 86, oxygen saturation (SpO_2_) 93% (room air), and blood pressure (BP) 110/60 mg. The patient was a current smoker with 88 pack‐years of smoking.

Blood laboratory data showed liver dysfunction, with aspartate aminotransferase, alanine transaminase, and alkaline phosphatase levels at 126, 137, and 910 IU/L, albumin was 2.7 g/dL, respectively. Viral hepatitis was excluded by lab data as HBs‐Ag(−), HBs‐Ag 0.01, HCV‐Ab(−), and HCV‐Ab 0.06. Patients had no history of drinking and alcoholic liver injury was ruled out. Antinuclear antibodies were negative and autoimmune hepatitis was excluded, and liver cirrhosis was excluded by the histology of needle biopsy that was luck of fattening of fibrosis, which will be described in detail later. The patient had no history of liver dysfunction. The carcinoembryonic antigen level was elevated to 730.5 ng/mL.

Whole‐body enhanced computed tomography (CT) revealed a 62 cm lung nodule in the right lower lobe in segment 9. Metastases to multiple lymph nodes (no. 10R, no. 7, no. 4, and no. 6) and bones. No liver tumor was detected.

Transbronchial lung biopsy (TBLB) of the lung nodule was performed. The patient was diagnosed with cStage IVB (cT3N3M1c) primary lung adenocarcinoma.

**FIGURE 1:**
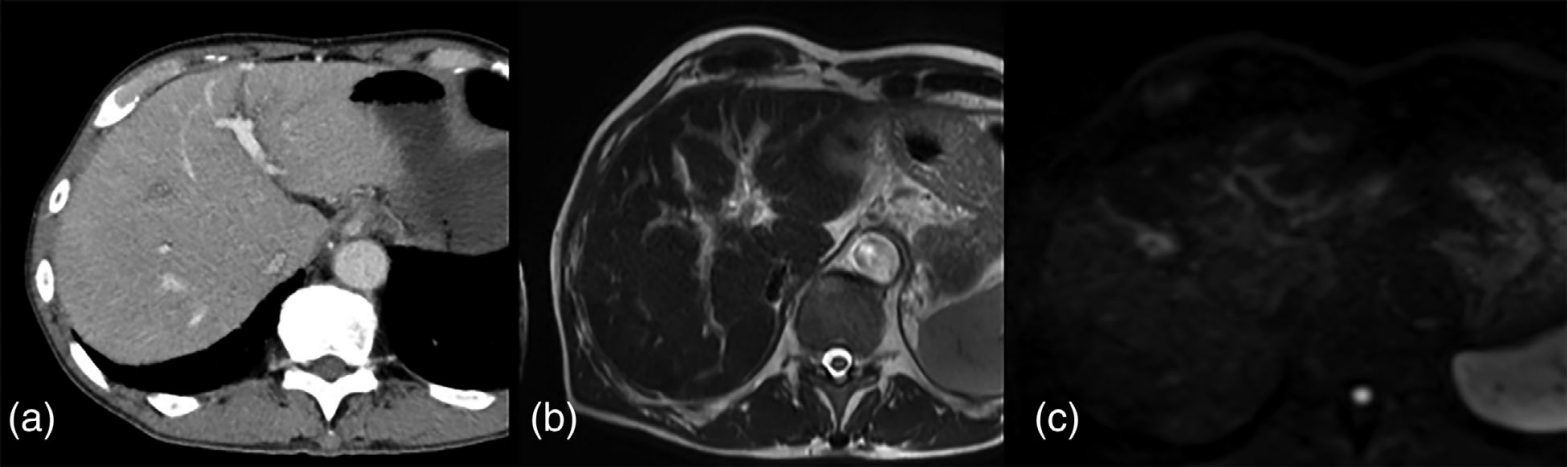
(a) The enhanced computed tomography (CT) image does not reveal a massive tumor, but low density in the periportal area can be detected. (b),(c) T_2_‐weighted and diffusion‐weight magnetic resonance imaging (MRI) shows high intensity at the periportal area

Abdominal ultrasonography detected a hypoechoic area around the portal vein. Dynamic enhanced CT scan of the portal phase revealed narrowing of the portal vein and a low‐density area surrounding the portal veins (Figure 1(a)). As a result of portal vein narrowing, a high‐density area associated with compensatory arterial blood flow increase was observed as a finding in the arterial phase. High‐signal intensity in the periportal area was demonstrated with T_2_‐weighted and diffusion‐weighted image of magnetic resonance imaging (MRI) (Figure 1(b),(c)). ^18^F‐FDG positron emission tomography (PET)/CT examination is an indispensable modality for lung cancer staging; however, PET/CT examination could not be performed because our hospital did not have the facilities, the patient's condition was poor, and we could not afford to go to another hospital to perform PET/CT.

Liver needle biopsy was performed by ultrasound‐guided from segment 5 of the right lobe of the liver. There was no target lesion and the puncture was blind. Immunostaining pattern of the cells were thyroid transcription factor 1 (TTF‐1)‐positive, napsin A‐positive, p53‐positive, and cytokeratin 7 (CK7)‐positive, which is suggestive of a metastatic liver tumor of lung adenocarcinoma.

Because of the poor general condition at admission, respiratory function tests were not performed throughout the course. CT showed no emphysema; however, it met grade 1 in modified British Medical Research Council questionnaire and clinically, it is presumed that COPD was present as a complication with lung cancer.

**FIGURE 2:**
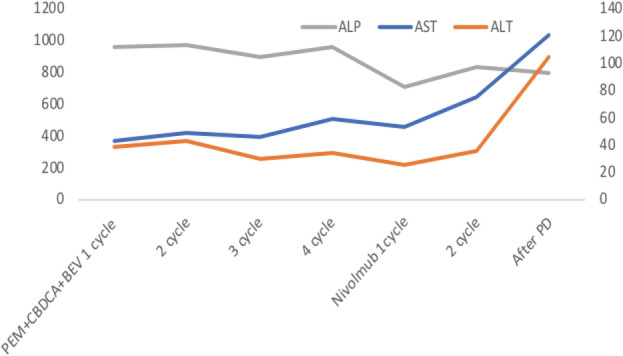
After the second line chemotherapy, liver enzymes increased and the condition worsened

**FIGURE 3:**
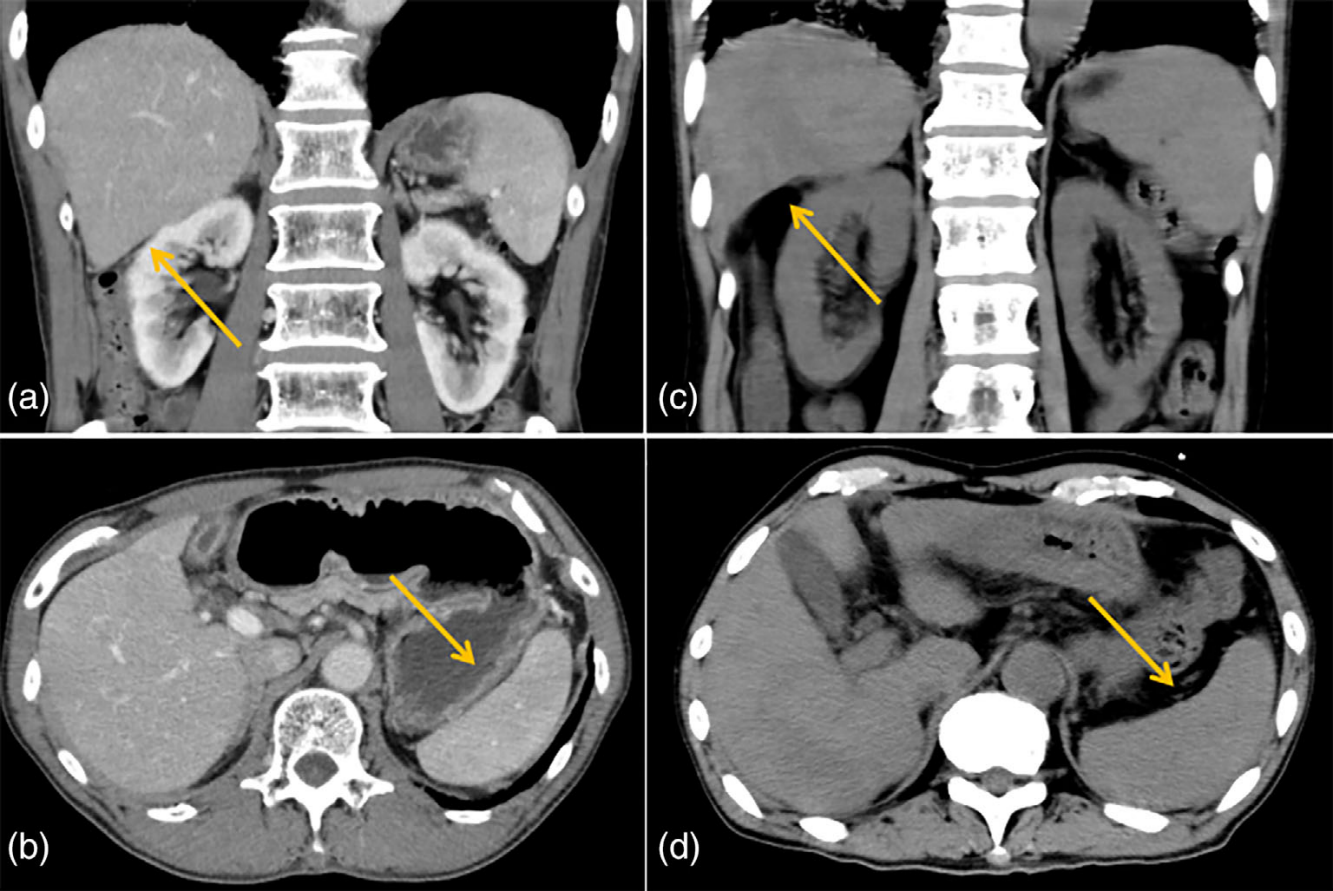
(a)–(d) Findings of liver atrophy and progression of splenomegaly were observed during the treatment

Pemetrexed plus carboplatin and bevacizumab were administered as the first‐line therapy for lung adenocarcinoma. The alkaline phosphatase levels declined after the administration of chemotherapy (Figure 2). After four cycles, the patient developed progressive disease. Nivolumab was administered as the second‐line therapy. After two cycles of administering nivolumab, there was exacerbation of the disease on the Eastern Cooperative Oncology Group Performance Status scale. CT confirmed liver atrophy and progression of splenomegaly during treatment (Figure 3(a),(b)). The patient died after worsening of liver failure.

An autopsy was performed. Lung adenocarcinoma was found in the lower lobe of the right lung, with a predominantly micropapillary pathological subtype. The gene mutations were all negative for ALK, EGFR, ROS1, BRAF and PDL1 was less than 1%. The liver was macroscopically hard and irregular.

**FIGURE 4:**
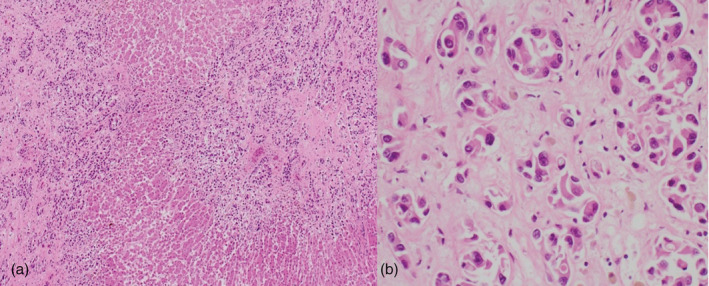
(a) The autopsy findings reveal the tumor cells infiltrating the central portal vein of the liver with hematoxylin and eosin (H&E) staining (×400 magnification). (b) The tumor cells appear lumpy with small glandular cavities with H&E staining (×200 magnification)

Microscopically, the liver was diffusely infiltrative replaced with tumor cells, in particular, tumor cell infiltration was strongly observed around the portal vein (Figure 4(a),(b)). In addition, infarction, fibrosis, and regenerated nodules were observed.

Gastric and esophageal varices, splenomegaly, and intraportal thrombus were present. A pathological diagnosis of pseudocirrhosis associated with diffuse metastasis of lung cancer was made, and the cause of death was determined to be portal thrombosis and liver failure associated with exacerbation of lung cancer. A comprehensive consent form was obtained from the patient at the time of admission, and after dissection, the family had permission to write a paper, and there are records.

## DISCUSSION

This case is extremely rare in two respects: (1) diffuse liver metastasis because of lung adenocarcinoma, and (2) pseudocirrhosis due to lung cancer.

Diffuse liver metastasis is common in gastrointestinal cancer or blood cancer, and there are few reports on its occurrence in lung cancer, particularly in small cell lung cancer.^7^ To our knowledge, this is probably the first report on diffuse liver metastasis in lung adenocarcinoma. Diffuse liver metastases are only visualized as slight abnormal findings around the portal vein on CT or MRI imaging,^8^ and caution is necessary while interpreting these findings. In patients with lung cancer who have abnormal findings around the portal vein with a hepatic disorder, drug‐induced hepatic disorder and viral hepatitis may be considered but these can be excluded based on the medical history of the patient, as in the present case. If differential diagnosis or determination of the etiology is difficult, it is necessary to perform a percutaneous liver biopsy without hesitation, as was done in this case. In this case, severe cancer infiltration and treatment resistance are considered to be associated with a history of severe smoking.^9^, ^10^

The concept of pseudocirrhosis was first reported by Honma^11^ as hepar lobtum. Although its pathophysiology is unclear, it is speculated that these changes result from liver metastasis itself, partly from drug‐induced liver injury and from scar contraction because of tumor shrinkage.^12^

Oliai et al.^2^ analyzed the clinical picture in breast cancer patients and reported that breast cancer patients with liver metastases who receive repeated chemotherapy tend to develop pseudocirrhosis.

In the present case, an autopsy was performed. There was diffuse liver metastasis in the background, and the growth of the tumor obstructed the central vein and portal vein, resulting in thrombosis and infarction of the liver parenchyma. The resulting progression of liver failure is presumed to be the cause of death. In addition, because thrombus formation and hepatic parenchymal infarction occurred in the subacute course, liver fibrosis occurred simultaneously. We consider that ischemia caused by vascular stenosis, including the portal vein caused by the tumor, contributed to pseudocirrhosis.

We have described a rare case of pseudocirrhosis and liver failure resulting from diffuse liver metastasis in lung adenocarcinoma. Even in lung adenocarcinoma, if liver damage with abnormal imaging findings around the portal vein are observed, diffuse liver metastasis can be suspected. A take‐away message from this report is that pseudocirrhosis can occur in lung cancer as well.

## CONFLICT OF INTEREST

The author declares that there is no conflict of interest.
